# The Week's Work

**Published:** 1911-08-05

**Authors:** 


### THE WEEK'S WORK.

### HOW THE CHARING CROSS APPEAL IS BEING RUN.

We hope that the general public realises the fact that the Charing Cross Hospital appeal is being pushed forward by an exhibit at the Festival of Empire now in progress at the Crystal Palace. Unfortunately it must be admitted that the appeal has suffered among other things by the distractions of Coronation year, and anything which tends to advertise that its promoters are still active must be supported by all who would like to see this hospital on its legs again. By one of those unfortunate chances which have beset this hospital from time to time its position in the Metropolis was just near enough to the Coronation processional route to make it seem a pity not to erect a stand, and at the same time not near enough to make practical a stand on anything like the same large scale that the other more fortunately placed hospitals were able to put up. Energy in the face of these difficulties is what is glaringly wanted, and we hope for the sake of Charing Cross Hospital that the Crystal Palace may prove an exceptionally popular place so long as the festival lasts. Praiseworthy trouble has been taken with the hospital's exhibit which includes those features most likely to attract the public—namely, a theatre, an $x$-ray demonstration room, a casualty room, and a hospital ward, carefully fitted up by the Hospitals and General Contracts Company. May this ward, busy as it is for the entertainment of the public, be the means of opening one at least of the closed wards at Charing Cross, which have a more permanent purpose to fulfil, that can be accomplished only by making this appeal the success which is still not impossible. Everyone is more or less coached now in hospital phraseology, thanks to the voluntary system, and the catalogue of this exhibit is a well thought out advertisement for the hospital and the organising firm of exhibitors, which should lead many of the public to send in subscriptions. They are wanted badly enough.

### MR. THIES' RECORD.

The farewell resolution to Mr. Conrad W. Thies which was passed by the Committee of Management of the Royal Free Hospital a few days ago, and which is to be engrossed and presented to him later, reminds hospital workers not only of his achievement, but that in four short weeks his quarter of a century of work for that institution comes to an end. It enables us also to publish a brief summary of his record, which it would have been premature to print when we announced his resignation, and which, we believe, will excite general interest. Mr. Thies has been so indefatigable a hospital secretary that many have not realised how closely he formerly identified himself with somewhat different interests, and the story of his attraction to hospital work, we believe, has never been told before. The bare facts are all that we have space enough to publish, but we propose next week to illuminate them with the flashlight of a personal impression from the point of view of one who worked under him and who knew him intimately enough to realise how interesting a circle of personalities was known by a man who struck the average hospital worker simply as a very able colleague. Mr. Thies has always been fond of travel, and his coming holiday, which begins in October, will be spent very largely abroad. On his return to England next spring, it is understood that he will devote some of his time to the work of the British Hospitals Association, of which, of course, he is honorary treasurer, and in the future usefulness of which he has great confidence. He also proposes to prepare an historical account of the Royal Free Hospital.

### HOSPITALS AND STATE AID.

Owing to press of matter the series of articles by our Special Commissioner on Hospitals and State Aid and on the German System has had to be interrupted. We hope, however, to resume the series in an early issue, when our Commissioner will deal specifically with the questions raised by correspondents with regard to several important points. Meanwhile we cordially thank hospital secretaries and workers who have favoured us with suggestions for future consideration.

### PROPOSED PAYMENT OF THE NEWCASTLE MEDICAL STAFF.

The resident medical staff at the Newcastle Royal Victoria Infirmary is asking the House Committee to pay £25 for each appointment of six months to all those members not in receipt of any payment at present. The classic arguments used are the expense of the medical training and the responsibility of the work, but it is not these that compel our attention. The reason at the back of the suggestion appears to be that for the past three months two of the appointments have been held by unqualified men, in spite of the fact that the vacancies were advertised in the leading medical journals. "The qualified men are thus compelled to take on their own shoulders the responsibility of the administration of anæsthetics by unregistered men." The suggestion is that the vacancies have not been filled because many qualified men cannot afford the expense of residence in the hospital without payment. A pound a week during the appointment (£25 for six months) is felt to be sufficient to overcome this difficulty, which is very common. It is interesting to note in this connection some remarks made last week by Mr. Jordan Lloyd in his "address in surgery." After referring to "the caprices of fashion and the demands of ritual among present-day operators," he urged with great emphasis the necessity for the payment of the honorary medical staffs. Discounting for the moment Mr. Lloyd's strong bias in favour of municipal or State-supported hospitals, we may note his argument that "the fifteen million of the public who are to be affected by the Insurance Bill will require active surgical treatment" on a scale so large as to necessitate the payment of the operators.

If the voluntary hospitals succeed in securing a payment *pro rata* for the new work to be thrust on them by the passing of this measure, the practitioners, as a necessary corollary, must be paid.

### OUT-PATIENT DEPARTMENT AT THE MIDDLESEX CLOSED.

Last Monday, according to an announcement by the Weekly Board, the out-patient department of the Middlesex Hospital was closed. It will remain closed until Monday, September 18, though we understand that accidents and urgent cases will be treated as usual. In the interval of closing certain repairs will be effected. It is to be hoped that no lay paper will make itself ridiculous by adversely criticising so obvious and reasonable a course. The attitude taken by some on the not dissimilar question of closing certain wards owing to the noise of building caused by the erection of Coronation stands by those hospitals on the route which were glad enough to have an opportunity to get their cleaning done at one time, makes this a hope instead of a certainty.

### QUEEN ALEXANDRA'S GIFT TO THE HOMŒPATHIC.

A portrait of King Edward with the words "King Edward VII." in Queen Alexandra's handwriting has been given by Her Majesty to the London Homœopathic Hospital. Col. Sir Arthur Davidson has sent instructions that Queen Alexandra desires the portrait to be placed in the King Edward VII. Memorial Ward, which forms part of the Sir Henry Tyler wing that was recently opened by H.R.H. Princess Louise. We hope that this Royal gift may stimulate the subscribers to complete the furnishing fund of the new wing.

### THE ALDERSHOT MEMORIAL.

The event of last week at Aldershot was the laying of the foundation-stone of a children's wing, which is being built as a memorial to King Edward. The ceremony was properly marked by the chief part being given to Mrs. Newcome, whose late husband had given the site. The new wing will increase the accommodation from twelve to some thirty beds, and Mr. W. T. Robertson, the Chairman of the Hospital Committee, together with the Honorary Secretary, Mr. W. Wren, are not unhopeful that the additional cost of maintenance will be forthcoming.

### HOSPITALS, THE INSURANCE BILL, AND THE LAY PRESS.

The gratifying and, on the whole, knowledgeable interest which the lay Press has taken in the hospital problem so far as it is affected by the Government's National Insurance Bill is a good sign of the awakened attention which the public in general has manifested in the hospital question as a whole. The excellent articles which have appeared in several London papers—notably in "Truth"—support the contentions we originally advanced in The Hospital when the subject of the position of our voluntary institutions under the Bill was receiving scanty attention. Those journals who differ from our conclusions do so on principles more than on details, and their case, briefly, is the case against the voluntary principle. On the other hand, those who accept the voluntary system, and desire to maintain it, realise to the full the difficulties which the Bill, if it passes the House of Commons without material modification of the clauses affecting the hospitals, will do to the voluntary hospitals. Meanwhile we are grateful to the lay Press for concentrating outside attention on the discussion, for such debate will at least expose some of the weak points in the arguments of extremists.

### THE WHITBY COTTAGE HOSPITAL.

Mr. J. G. Tomlinson is to be congratulated on the support which, partly through his efforts, has been obtained by the Whitby Cottage Hospital, at Grape Lane, Yorkshire. Since his appointment as honorary secretary about four years ago, a local Hospital Football Cup has been started, which has handed over no less than £60 to the institution. The funds of cottage hospitals often require a good deal of trouble to improve in this way, and we are not surprised that the honorary treasurer and assistant secretary have been recently re-elected to their posts in the persons of Mr. G. E. Todd and Mr. W. J. Tyreman. The future of this institution, to anyone conversant with the district, is one of interest in that the cottage hospital of to-day sometimes becomes the big institution of the future.

### THE MAHARAJA OF GWALIOR AND KING EDWARD'S FUND.

The fact that his Majesty has allotted to the King's Fund one quarter of his Highness's the Maharaja of Gwalior's Coronation gift of £8,000 will call our attention to this well-known potentate who has taken a great interest in institutional charities in India. The Maharaja has identified himself in the most practical way with hospital work on at least one famous occasion. In China during the Boxer war he was serving on the staff of General Sir Alfred Gaselee, and that experience led him to present a hospital ship for the accommodation of the wounded. The confidence which the King's Fund inspires all over the world could hardly be better illustrated than by its name being placed first in the list of charities chosen for a special benefit by his Majesty King George. It was because we realised the importance of maintaining that confidence that we ventured to criticise the Fund's Memorandum on the Insurance Bill two weeks ago. When almost everyone was hedging, the King's Fund should have used its well-deserved authority to speak out without vacillation.

### THIS WEEK'S DRUG MARKET.

Business in the drug market continues quiet, but opium continues to attract attention; the price of this drug still tends to advance and in consequence morphine is higher in value. Notwithstanding the fact that the yield of cod liver oil from this season's catch of cod in Norway is larger than that of last season the price is firmly maintained for the present; buyers should be cautious, however, as judging from the statistical position of the oil it is not improbable that prices will recede in due course. Jalap is tending somewhat higher in price. Ipecacuanha is rather cheaper. Essence of lemon is dearer and a good business has been done in citric acid.

